# Stimuli-Responsive Assemblies for Sensing Applications

**DOI:** 10.3390/gels2010008

**Published:** 2016-02-15

**Authors:** Xue Li, Yongfeng Gao, Michael J. Serpe

**Affiliations:** Department of Chemistry, University of Alberta, Edmonton, AB T6G 2G2, Canada; xue13@ualberta.ca (X.L.); yg2@ualberta.ca (Y.G.)

**Keywords:** stimuli-responsive polymers, photonic materials, poly (*N*-isopropylacrylamide)-based microgels, etalons, optical sensing

## Abstract

Poly (*N*-isopropylacrylamide) (pNIPAm)-based hydrogels and hydrogel particles (microgels) have been extensively studied since their discovery a number of decades ago. While their utility seems to have no limit, this feature article is focused on their development and application for sensing small molecules, macromolecules, and biomolecules. We highlight hydrogel/microgel-based photonic materials that have order in one, two, or three dimensions, which exhibit optical properties that depend on the presence and concentration of various analytes. A particular focus is put on one-dimensional materials developed in the Serpe Group.

## 1. Introduction

Polymer-based stimuli responsive materials have been of great interest over the years due to their ability to convert external chemical and/or physical stimuli into observable changes of the material itself [1,2,3]. Some of the most important of these materials are responsive hydrogels, which are hydrophilic crosslinked polymer networks capable of changing their solvation state in response to various stimuli [4,5,6]. Hydrogel particles (e.g., microgels) can also be synthesized, and typically have diameters of 100–2000 nm [7,8]. Over the years, many polymers, and polymer-based materials, have been identified that exhibit a specific response to a variety of stimuli. Some of those stimuli include temperature [9,10], light [11,12], electric [13], and magnetic fields [14]. These responsivities make hydrogels very useful for many applications, e.g., sensing [15,16], drug delivery [17,18], artificial muscles [19,20], tissue engineering [21,22], and self-healing materials [23,24]. Among these, thermoresponsive materials have been the most extensively studied, and poly (*N*-isopropylacrylamide) (pNIPAm)-based hydrogels and microgels are the most well-known and extensively studied thermoresponsive materials [25,26,27]. PNIPAm is fully soluble in water below ~32 °C, and transitions to an “insoluble” state when the temperature is above 32 °C. This transition is observed as a coil-to-globule transition, where the polymer transitions from an extended to collapsed state, respectively [28]. The conformational change is also accompanied by a water exchange process. That is, when pNIPAm undergoes the coil-to-globule transition, water is “expelled”, while water is “absorbed” when pNIPAm undergoes a globule-to-coil transition. Similarly, crosslinked pNIPAm-based hydrogels and microgels contract upon heating, and swell with water upon cooling [29,30,31]. This swelling-deswelling transition is fully reversible over multiple heating/cooling cycles. 

While there are many uses of pNIPAm hydrogels and microgels, a majority of this review focuses on their use for sensing applications. Specifically, this review focuses on the use of microgels and hydrogels as components of photonic material (PM) assemblies. A specific example of a PM is a photonic crystal (PC); PCs are composed of materials of varying refractive indices arranged in an ordered fashion in one, two, or three dimensions (1D, 2D, 3D). There are many examples of PCs in nature, most commonly associated with the vibrant colors of butterfly wings and the opal gemstone. These materials are unique because, unlike many other colored materials found in nature that exhibit color due to the absorbance of light by small molecule chromophores, PCs are colored due to their structure. Specifically, the opal gemstone is composed of a close-packed array of colloids (typically silica), which are capable of interacting with wavelengths of light in the visible region of the electromagnetic spectrum. These interactions lead to constructive and destructive interference of the light in the assembly, leading to specific wavelengths of light being reflected, which leads to the observed color [32]. A major goal of many research groups around the world is to generate synthetic colloidal crystals. This is typically done by “forcing” colloids of high refractive index into an ordered array in a matrix of relatively low refractive index (e.g., air, water, polymer). If the particle periodicity (*i.e.*, refractive index periodicity) is on the order of visible wavelengths of light, then the device will appear colored. This is a direct result of light refraction, reflection, and diffraction off the material’s particles, which leads to light interference, and hence color [32,33,34]. PM and PCs are of great interest for various applications, including optics [35,36], actuators [37,38], sensors [39], controlled drug delivery [40] and for display devices [41,42]. 

In this submission, we will first discuss examples of PMs generated from inorganic components (such as silica particles) and block copolymers, and 1D, 2D, and 3D PCs fabricated from them. Then we will discuss their use for sensing and biosensing, with a particular focus on 1D PCs constructed by our group from pNIPAm-based microgels.

## 2. Photonic Materials

**Figure 1:**
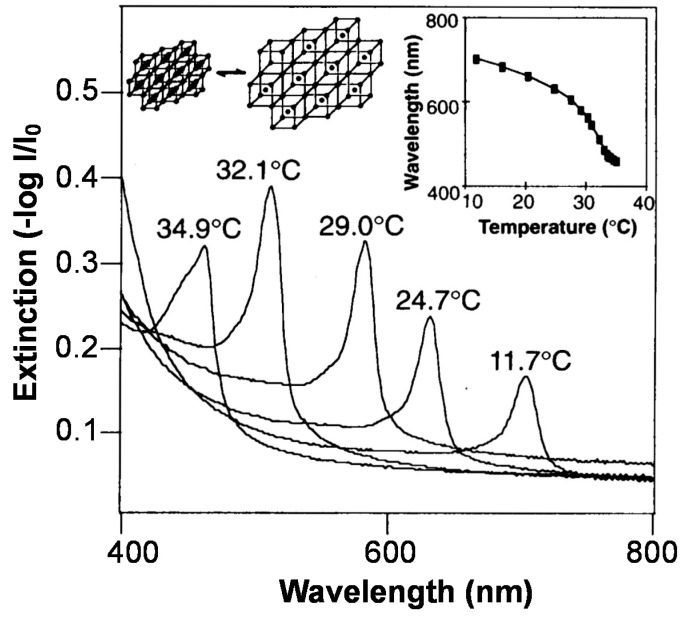
Temperature tuning of Bragg diffraction from a 125-µm-thick polymerized crystalline colloidal array (PCCA) film of 99-nm polystyrene (PS) spheres embedded in a poly (*N*-isopropylacrylamide) (pNIPAm) gel. The shift of the diffraction wavelength results from the temperature-induced volume change of the gel, which alters the lattice spacing. Spectra were recorded in a UV-visible-near IR spectrophotometer with the sample placed normal to the incident light beam. The inset shows the temperature dependence of the diffracted wavelength for this PCCA film when the incident light is normal to the (110) plane of the lattice. Reprinted with the permission from [43] Copyright ^©^ 1996, American Association for the Advancement of Science.

As discussed above, the opal gemstone is composed of particles packed into an ordered array—these structures are sometimes referred to as colloidal crystal arrays (CCAs). Both natural and synthetic CCAs exist, and can yield extremely colorful materials. The color the materials exhibit depends on the spacing between the array elements (and other parameters), according to Equation (1):

(1) *m*λ= 2*nd*·sin θ

where *m* is the order of diffraction, λ is the wavelength of incident light, *n* is the refractive index of the optical components, *d* is the interplanar spacing, and θ is the angle between the incident light and the diffracting crystal planes, which are oriented parallel to the crystal surface in the prepared CCA. Since the color the material exhibits depends directly on the array element spacing, expansion/contraction of responsive polymers coupled to CCAs can be used to tune the spacing, and hence the color of the materials. One of the most extensively used responsive polymer for this purpose is pNIPAm, and early examples from the Asher Group showed that the volume changes that pNIPAm undergoes as a function of temperature can be used to tune the visual color of colloidal crystals. The Asher Group [43] showed that the optical properties of these materials, referred to as polymerized crystalline colloidal arrays (PCCAs), could be tuned quite dramatically with temperature. Specifically, as shown in Figure 1, the Bragg peak could be tuned between 704 and 460 nm by variation in the temperature.

**Figure 2:**
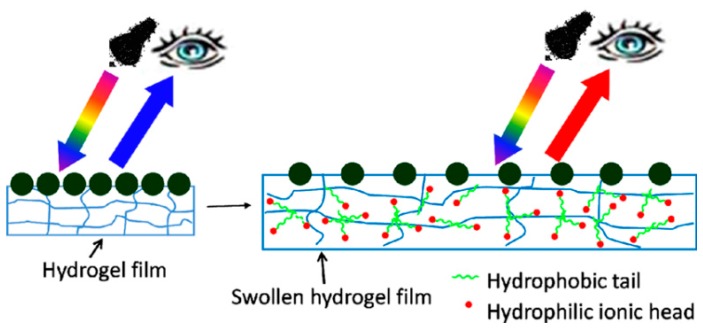
Schematic illustration of a 2D photonic crystal sensor formed by polymerization of a pNIPAm hydrogel network onto a 2D array of 490 nm PS particles, which was added to a liquid Hg surface. The pNIPAm hydrogel swells upon binding of surfactant molecules. The 2D particle spacing increases upon swelling of the pNIPAm hydrogel, red shifting the diffracted wavelength. Reprinted with the permission of [46] Copyright ^©^ 2012, American Chemical Society.

**Figure 3:**
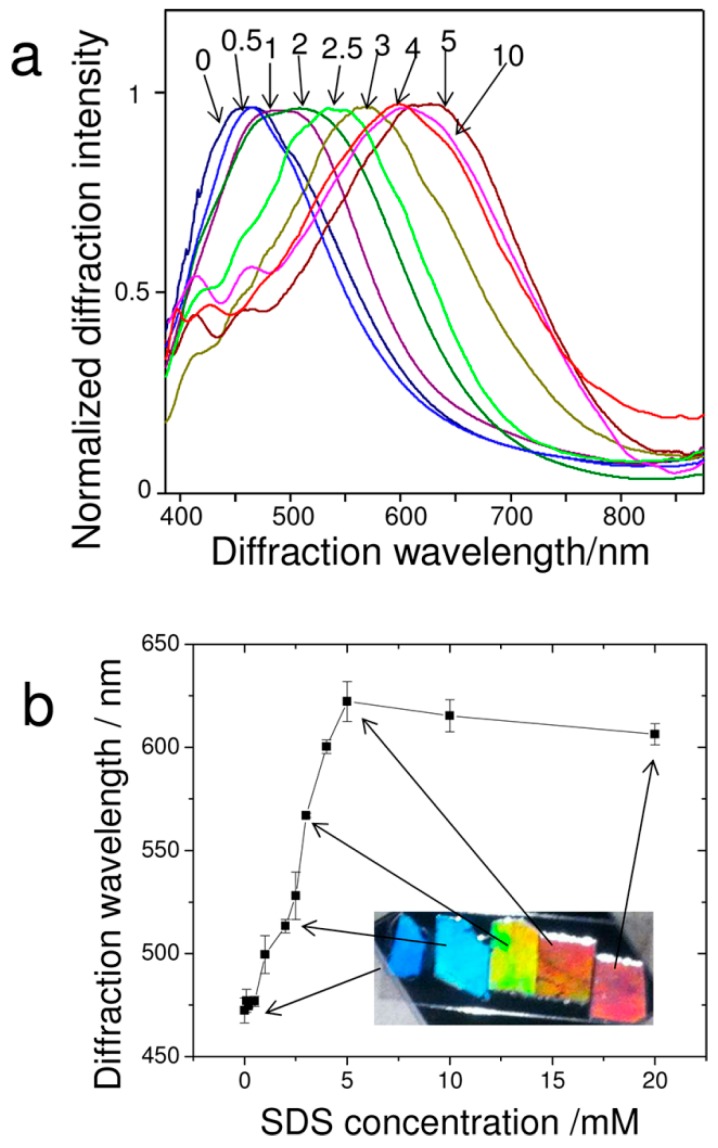
(**a**) Normalized and smoothed diffraction spectra of 2D pNIPAm-based sensors at different concentrations of aqueous sodium dodecyl sulfate (SDS) solutions. Each number in the graph corresponds to the different concentration in the unit of mM. The measurement angle between the probe and the normal to the 2D array was 28°; (**b**) Wavelength of diffracted peak *versus* SDS concentration. The inset shows photographs taken close to the Littrow configuration at an angle of 28° between the source and camera to the 2D array normal. Reprinted with the permission of [46] Copyright ^©^ 2012, American Chemical Society.

In many subsequent investigations, Asher and coworkers entrapped CCAs in various hydrogel materials, which changed volume (and hence optical properties) in the presence of various analytes, and upon exposure to a variety of stimuli [6,44,45]. In a more recent example, the Asher group developed a novel two-dimensional (2D) CCA for the visual detection of amphiphilic molecules in water [46]. These 2D photonic crystals were placed on a mirrored surface (liquid Hg), and exhibited intense diffraction that enabled them to be used for detection of analytes by observation of visual color changes. Figure 2 shows a schematic illustration of the 2D photonic crystal. A monolayer of 2D close-packed polystyrene (PS) particles was embedded in a pNIPAm-based hydrogel film. Binding of surfactant molecules increased the charge density in the hydrogel. This resulted in a swelling of the pNIPAm-based hydrogel, and a concomitant change in the distance between the array elements. The resulting increase in particle spacing red shifts the 2D diffracted light according to Equation (2):

(2) *m*λ = 3^1/2^ × *d* sin θ

where *m* is the diffraction order, λ is the wavelength of the diffracted light, *d* is the nearest neighboring particle spacing, and θ is the angle between the incident light and the normal to the 2D array. For a fixed angle of incidence (θ), the diffracted wavelength (λ) is proportional to the 2D particle spacing (*d*). As shown in Figure 3, normalized and smoothed diffraction spectra of 2D pNIPAm-based sensors were obtained at different concentrations of aqueous, sodium dodecyl sulfate (SDS) solutions and diffraction wavelengths *versus* different concentration of SDS. 

**Figure 4:**
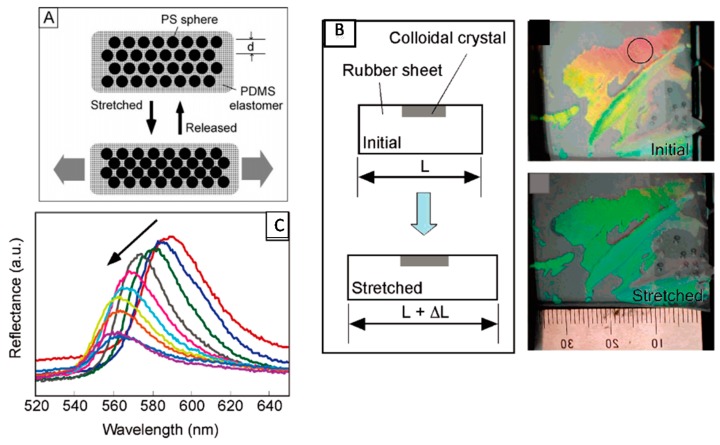
(**A**) Reversible tuning by stretching of the lattice distance of a PS colloidal crystal embedded in a poly(dimethylsiloxane) (PDMS) elastomer matrix; (**B**) Changes in the structural color of the colloidal crystal film covering the silicone rubber sheet; (**C**) Relationship between the reflectance peak position and elongation of the silicone rubber sheet by stretching. The peak position shifted from 590 to 560 nm, and the reflectance intensity decreased gradually as indicated by the arrow. Reprinted with the permission of [47] Copyright ^©^ 2005, American Chemical Society.

In another example, Fudouzi and coworkers developed an elastic poly(dimethylsiloxane) (PDMS) sheet with a thin layer of cubic close packed polystyrene particles embedded. This material exhibited structural color, which could be tuned as a function of the extent of stretching [47]. Specifically, Figure 4A shows that the sheet can be stretched “horizontally”, which decreases the size of the material in the vertical direction leading to the decrease in the lattice spacing of (111) planes of the array, resulting in a blue shift of the device’s reflectance peaks and a concomitant visual color change, as shown in Figure 4B,C. The ability of these materials to change color as a function of PDMS elongation makes them well suited for quantifying mechanical strains on materials. Ultimately, this could be used for quantifying the fidelity of structures such as buildings and bridges.

**Figure 5:**
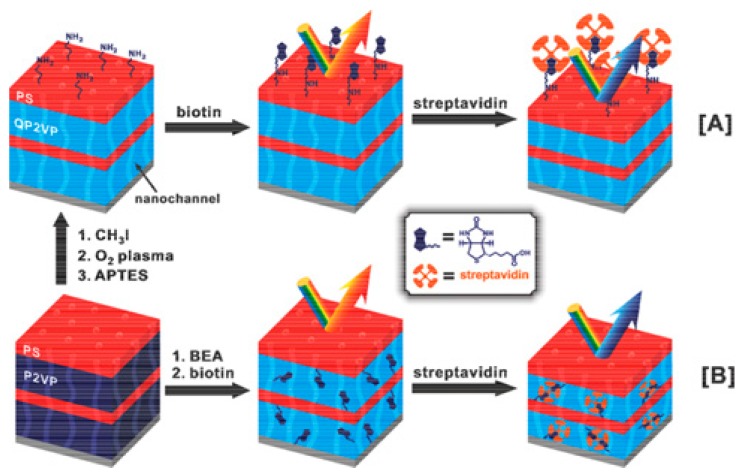
Preparation and use of biotinylated photonic gels. Procedure used for biotinylating the (**A**) surface of photonic gel films, and (**B**) the inside of photonic gel films. Reprinted with the permission of [48] Copyright ^©^ 2012, The Polymer Society of Korea and Springer Science + Business Media Dordrecht.

**Figure 6:**
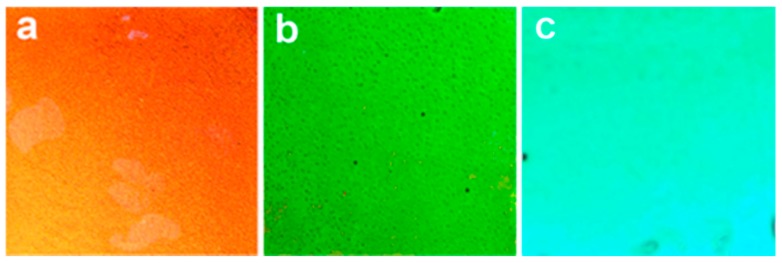
Color changes of the photonic gels with inside biotinylation in response to streptavidin; (**a**) [S] = 0 M; (**b**) [S] = 0.5 Mm; (**c**) [S] = 10 mM. Reprinted with the permission of [48] Copyright ^©^ 2012, The Polymer Society of Korea and Springer Science + Business Media Dordrecht.

Recently, there has been a growing interest in using block copolymer-based photonic gels for sensing and biosensing applications. Block copolymers offer the flexibility of fabricating 1D, 2D, and 3D photonic materials through self-assembly, making them relatively easy to fabricate. Some photonic gels are extremely sensitive to a change in charge and/or charge density in the gel matrix, as well as the dielectric environment. Proteins are highly charged dielectric materials, and thus the electrostatic and dielectric environment of photonic gels can change abruptly upon protein binding. This property has been harnessed by modifying the PCs with molecules that can bind specific targets, e.g., proteins, and the change in the local environment upon binding of the target can change the spacing between the material’s array elements. In one example, Kang and coworkers [48] have generated PCs using the ability of polystyrene-b-quaternized poly(2-vinyl pyridine) (PS-b-QP2VP) to self-assemble into a photonic gel, and modified the structure with biotin. This is shown schematically in Figure 5A,B for the “on-gel” and “in-gel” configurations they investigated. Upon binding streptavidin the spacing of the array elements changed, and a visual color change with streptavidin binding was observed for the in-gel photonic materials, as shown in Figure 6.

## 3. PNIPAm Microgel-Based 1D PCs and Their Application for Sensing and Biosensing

**Figure 7:**
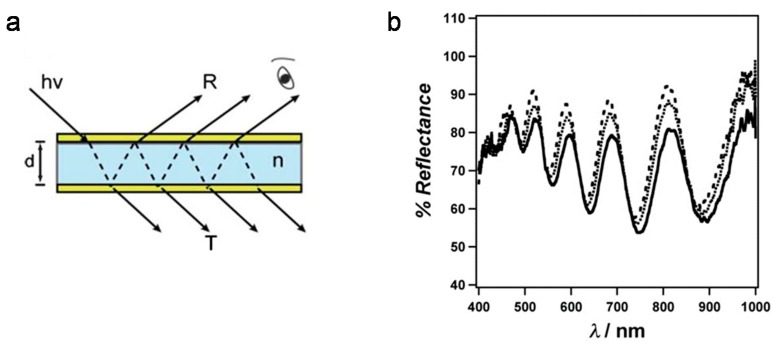
(**a**) Structure of the microgel-based etalon. Two reflective metal layers (in this case Au, top and bottom layers) sandwiching a dielectric (middle); (**b**) Representative reflectance spectra from a pNIPAm microgel-based etalon. Reprinted with the permission of [54] Copyright ^©^ 2012, Royal Society of Chemistry.

Compared with other pNIPAm microgel-based photonic materials, which exhibit order in 2D or 3D [46,49,50], the Serpe Group discovered color tunable materials (etalons) that exhibit structure in 1D. This was accomplished by sandwiching a pNIPAm microgel-based layer between two thin Au layers, which act as mirrors. The devices exhibit visual color, and unique multipeak reflectance spectra—the position of the peaks in the reflectance spectra primarily depend the thickness of the microgel layer, according to Equation (3),

(3) *m*λ = 2*nd*·cosθ

where *m* is the peak order, *n* is the refractive index of the dielectric material, *d* is the distance between Au layers, and θ is the angle of incidence [51,52]. The structure of the device and representative reflectance spectra are shown in Figure 7. Since the optical properties primarily depend on the thickness of the microgel layer (controls the mirror-mirror distance), the microgel’s response to a stimulus results in changes in the optical properties [53,54,55,56,57,58,59]. This property is extremely important for sensing applications, since the solvation state of microgels can be made to depend on many different stimuli. 

**Figure 8:**
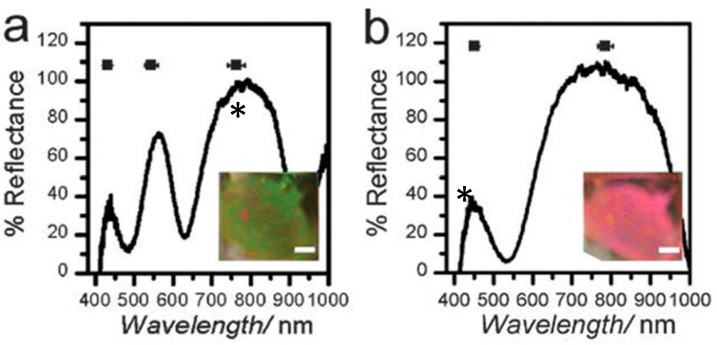
Representative spectra for an etalon in pH 3 solution (2 mM ionic strength (I.S.), I.S. adjusted with NaCl) at (**a**) 25 °C, (**b**) 37 °C. The star labelled peak is for the same reflection order from the device. The insets show the corresponding photographs of the devices. Reprinted with the permission of [60] Copyright ^©^ 2013, Royal Society of Chemistry.

The most basic response of the devices is to temperature, due to the collapse of the microgels in water at *T* > 32 °C. This leads to a decrease in the thickness of the microgel layer, and hence Equation (3) predicts a blue shift in the device’s reflectance peaks. As can be seen in Figure 8, the device’s reflectance peaks show a blue shift (e.g., the star-labelled peaks) with increasing temperature in aqueous solution. This is due to microgel deswelling, resulting in the decrease of the distance between the device’s Au layers [60].

**Figure 9:**
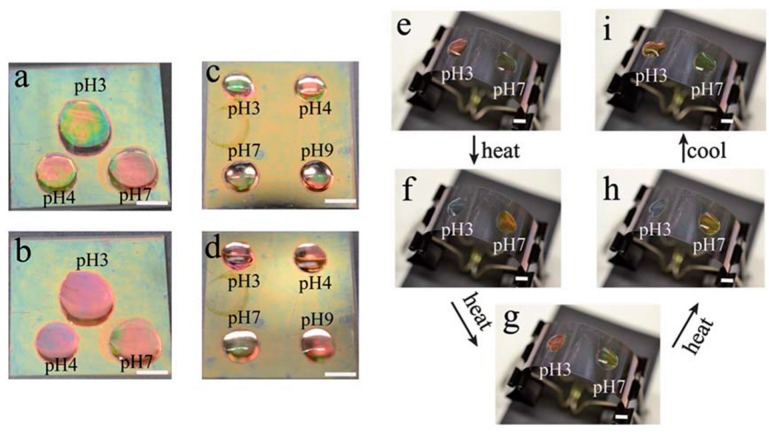
Photographs of an etalon with solutions of various pH spotted on a single surface (**a**, **c**, **e**, **i**) 25 °C and (**b**, **d**, **f**, **g**, **h**) 37 °C; (**f**) ~3 min after heating; (**g**) ~5 min after heating; (**h**) ~6 min after heating. In each panel, the scale bar is 5 mm. Reprinted with the permission of [54] Copyright ^©^ 2012, Royal Society of Chemistry.

By copolymerizing acrylic acid (AAc) (pK_a_ ~ 4.25) with NIPAm, pH responsive pNIPAm-co-AAc microgels and microgel-based etalons could be generated. The pH responsivity is a result of Coulombic repulsion and osmotic swelling in the microgel layer at pH > pKa as a result of the charges on the deprotonated AAc groups. Furthermore, we sought to determine if spatially isolated regions of pNIPAm-co-AAc microgel-based etalons can be independently modulated [54]. Figure 9 shows an etalon with solutions of different pH spotted on spatially isolated regions. As can be seen, the spots appear different colors, which can be changed as a function of temperature and pH, independently. In another example, we have shown that pNIPAm-co-AAc microgel-based etalons could be attached to the surface of a quartz crystal microbalance, and used for the very sensitive detection of solution pH [55,61]. 

**Figure 10:**
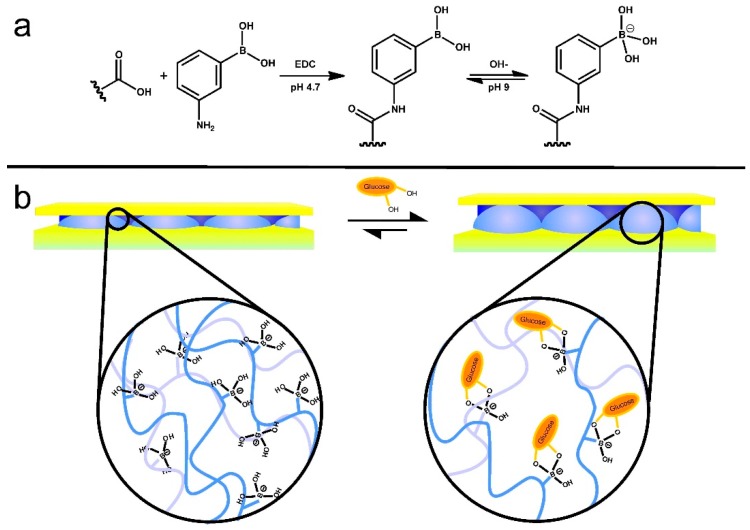
(**a**) Reaction scheme for the functionalization of the acrylic acid moieties on the microgel with 3-aminophenylboronic acid (APBA) followed by the activation of the boronic acid at high pH and (**b**) a cartoon depiction of the glucose responsivity of an APBA functionalized microgel etalon at pH 9. Reprinted with the permission of [53] Copyright ^©^ 2012, Springer Verlag.

Microgel-based etalons were also fabricated that could detect the concentration of glucose in solution. This was done by fabricating 3-aminophenylboronic acid (APBA) functionalized microgels [53]. As illustrated in Figure 10, glucose binding with the boron atom will promote more boron atoms to become negatively charged resulting in swelling of the microgel layer. Because λ is proportional to the distance between the two mirrors, *d*, the swelling of microgels gives rise to a red shift of the reflectance peaks. 

**Figure 11:**
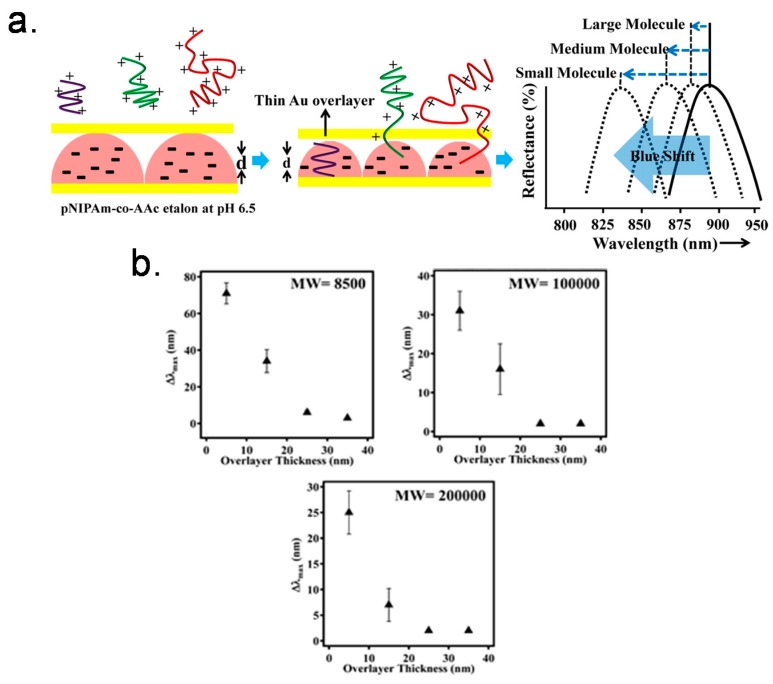
(**a**) Schematic depiction of polyelectrolyte penetration through the porous Au overlayer of an etalon and a schematic of the anticipated response; (**b**) Shift of λ_max_ for pNIPAm-co-AAc etalons in pH 6.5 after addition of pDADMAC solution with MW 8500, <100,000, and 100,000−200,000. Reprinted with the permission of [62] Copyright ^©^ 2013, American Chemical Society.

Etalons composed of either pNIPAm-co-AAc or pNIPAm-co-*N*-(3-aminopropyl)methacrylamide hydrochloride (pNIPAm-co-APMAH) microgels were also constructed [62]. We investigated their response to the presence of linear polycations and/or polyanions. When the etalon was at a pH that renders the microgels multiply charged, the microgel layer of the etalon deswells in the presence of the oppositely charged linear polyelectrolyte; it is unresponsive to the presence of the like charged polyelectrolyte. Furthermore, the etalon’s response depended on the thickness of the Au overlayer. For example, low molecular weight (MW) polyelectrolyte could penetrate all Au overlayer thicknesses, while high MW polyelectrolytes could only penetrate the etalons fabricated from thin Au overlayers, as shown in Figure 11a. We hypothesize that this is due to a decrease in the Au pore size with increasing thickness, which excludes the high MW polyelectrolytes from penetrating the microgel-based layer. This is supported by other investigations conducted in the group [63]. Figure 11b shows the shift of λ for pNIPAm-co-AAc etalons in pH 6.5 solution after addition of poly (diallyldimethylammonium chloride) (pDADMAC) solution of different molecular weights (MW). From this observation, we then developed pNIPAm microgel-based etalons and etalon arrays to determine the molecular weights of polymers in solution [64]. These devices show promise as MW selective sensors and biosensors. 

**Figure 12:**
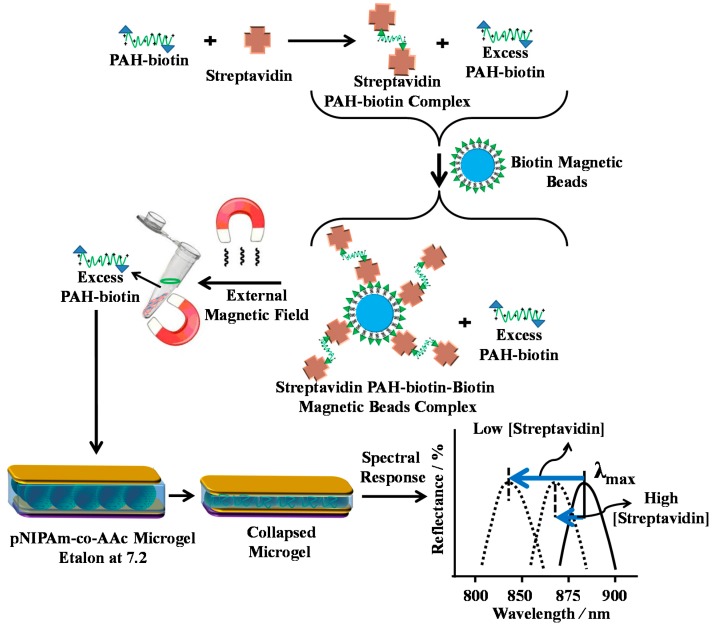
Streptavidin (the analyte) is added to an excess amount of biotin-modified poly (allylamine hydrochloride) (PAH). The streptavidin–biotin–PAH complex is then removed from solution using biotin modified magnetic particles, leaving behind free, unbound PAH. The unbound PAH is subsequently added to a pNIPAm-co-AAc microgel-based etalon immersed in aqueous solution at a pH that renders both the microgel layer and the PAH charged. As a result, the etalon’s spectral peaks shift in proportion to the amount of PAH–biotin that was added. This, in turn can be related back to the original amount of streptavidin added to the PAH–biotin. Reprinted with the permission of [65] Copyright ^©^ 2013, Elsevier.

**Figure 13:**
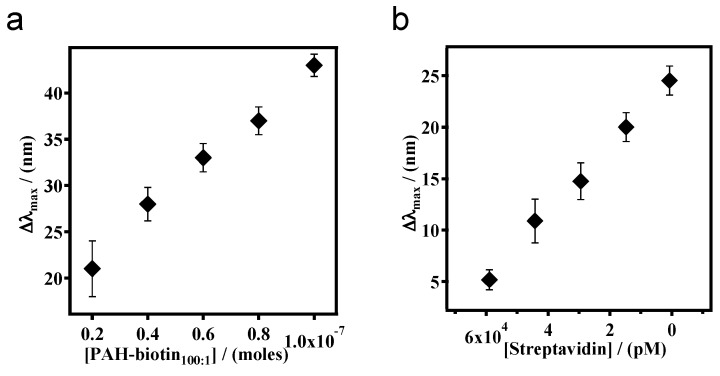
Cumulative shift of the etalon’s *m* = 3 reflectance peak with different amounts of (**a**) PAH-biotin_100:1_ (**b**) streptavidin added to PAH–biotin_100:1_. The pNIPAm-co-AAc microgel-based etalon was soaked in pH 7.2 throughout the experiment, while the temperature was maintained at 25 °C. Each point represents the average of at least three independent measurements, and the error bars are standard deviation for those values. Reprinted with the permission of [65] Copyright ^©^ 2013, Elsevier.

In an effort to exploit this observed phenomenon, we reported that biotinylated polycationic polymer can penetrate through the Au overlayer of a pNIPAm-co-AAc microgel-based etalon and cause the microgel layer to collapse [56,65]. The collapse results in a shift in the spectral peaks of the reflectance spectra. We found that the extent of peak shift depends on the amount of biotinylated polycation added to the etalon, which can subsequently be used to determine the concentration of streptavidin in solution at nM-pM concentrations. The sensing mechanism is shown schematically in Figure 12. As shown in Figure 13, the blue shift in the spectral peaks depends linearly on the amount of streptavidin, and hence PAH-biotin, added to the etalon. We were able to detect streptavidin concentrations in the nM range without any system optimization. We also note that this response is unique due to the fact that the response is highest for the lowest streptavidin concentration—this is counterintuitive based on the fact that other analytical approaches show small responses for low analyte concentrations.

**Figure 14:**
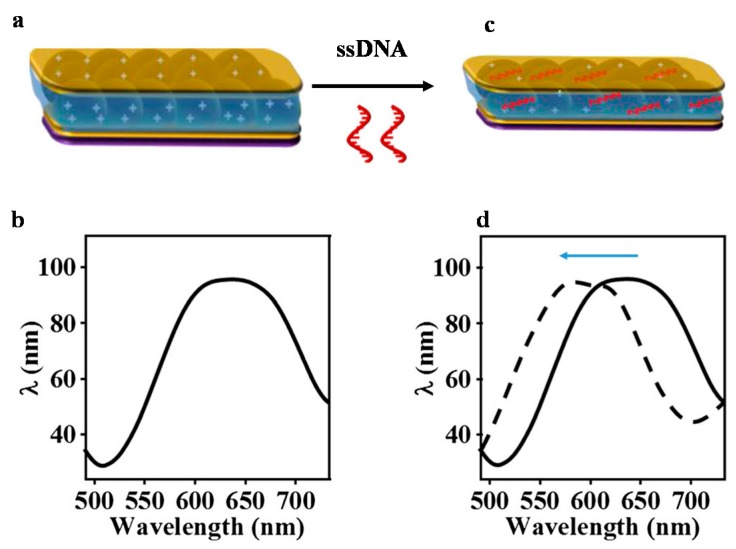
(**a**) The basic structure of pNIPAm-co-*N*-(3-aminopropyl) methacrylamide hydrochloride microgel-based etalon; (**b**) A schematic representation of a single reflectance peak. Here, the microgels are positively charged in water with pH < 10.0; (**c**) After addition of ssDNA, the microgels were crosslinked and collapsed, reducing the distance between the two Au layers of the device; (**d**) The peak of the reflectance spectrum shifts to a shorter wavelength. Reprinted with the permission of [57] Copyright ^©^ 2014, Springer Verlag Berlin Heidelberg.

**Figure 15:**
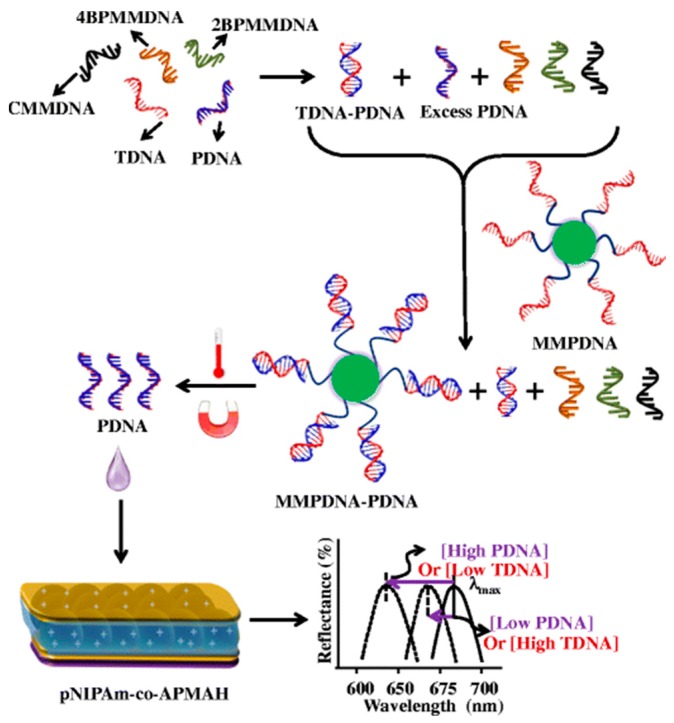
The protocol used for indirectly sensing target DNA (TDNA), by sensing probe DNA (PDNA). In this case excess PDNA can be related to TDNA concentration, even in the presence of DNA that is completely noncomplementary to PDNA (CMMDNA), and with 2 and 4 base pair mismatches (2BPMMDNA and 4BPMMDNA). Reprinted with the permission of [57] Copyright ^©^ 2014, Springer Verlag Berlin Heidelberg.

Many groups have investigated novel stimuli-responsive polymer-based biosensing systems for detection of proteins and DNA [66,67,68]. As mentioned above, pNIPAm based microgels can be modified with a variety of functional groups that can be incorporated into our optical devices to sense various targets, such as glucose, proteins, and DNA [53,56]. For example, poly (*N*-isopropylacrylamide-co-*N*-(3-aminopropyl) methacrylamide hydrochloride) (pNIPAm-co-APMAH) microgels were synthesized via temperature ramp, surfactant-free, free radical precipitation polymerization [57,69]. PNIPAm-co-APMAH microgels are positively charged at pH 7.2, and when negatively charged DNA is added to the etalon composed of these positively charged microgels, the microgels are crosslinked and collapse due to electrostatics and the devices exhibit a spectral shift, as shown in Figure 14. The sensing protocol that we developed from this phenomenon is shown in Figure 15. As can be seen in Figure 15, an excess amount of PDNA is exposed to a solution containing TDNA and DNA with a completely mismatched sequence (CMMDNA), and DNA with four (4BPMMDNA) and two base mismatches (2BPMMDNA). The PDNA binds the TDNA completely, leaving behind excess, unbound PDNA in solution. Magnetic microparticles (MMPDNA) that are functionalized with the complete complement to PDNA were added to the solution to capture the excess PDNA. A magnet was then used to isolate the magnetic microparticles bound with PDNA (MMPDNA–PDNA) from the solution. After washing the MMPDNA–PDNA, the PDNA was recovered by heating the solution to melt the DNA off of the MMPDNA–PDNA, and the excess PDNA was recovered and added to the etalon. In this case, a large spectral shift from the etalon corresponds to a large excess of PDNA, which means a low concentration of TDNA was present in the initial solution. The opposite is true as well—a low concentration of PDNA left in solution yields a small spectral shift from the device, meaning there was a large amount of TDNA present in the initial solution. This illustrates the strength of the current system—low concentrations of TDNA yield large spectral shifts making the device more sensitive to low DNA concentrations.

**Figure 16:**
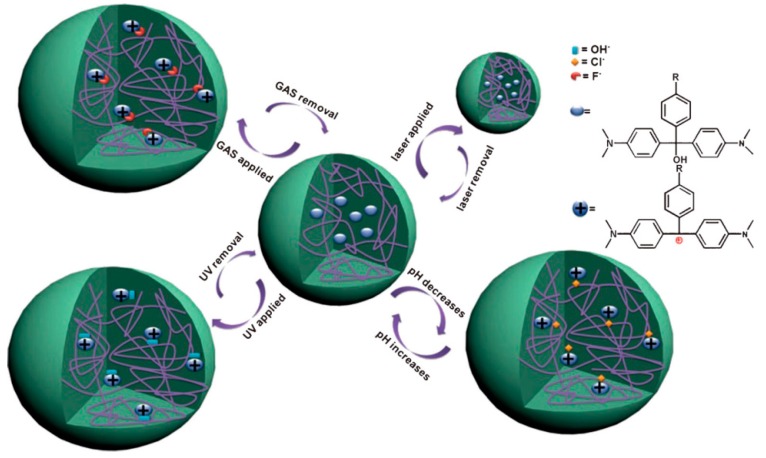
The various responses expected from TPL-modified microgels. The TPL structure is shown on the right. Reprinted with the permission of [58] Copyright ^©^ 2014, WILEYVCH Verlag GmbH & Co. KGaA, Weinheim.

Recently, our group developed novel multiresponsive pNIPAm-based microgels by incorporation of the molecule triphenylmethane leucohydroxide (TPL) into their structure. Figure 16 shows the schematic depiction of TPL-modified microgels and their response to various stimuli. These microgels were subsequently used to fabricate etalons, and the optical properties investigated in response to ultraviolet and visible irradiation, solution pH changes, and the presence of a mimic of the nerve agent Tabun was characterized [58]. We also clearly showed that the optical properties of the device depended dramatically on these stimuli and show great promise for remote actuation and sensing. 

**Figure 17:**
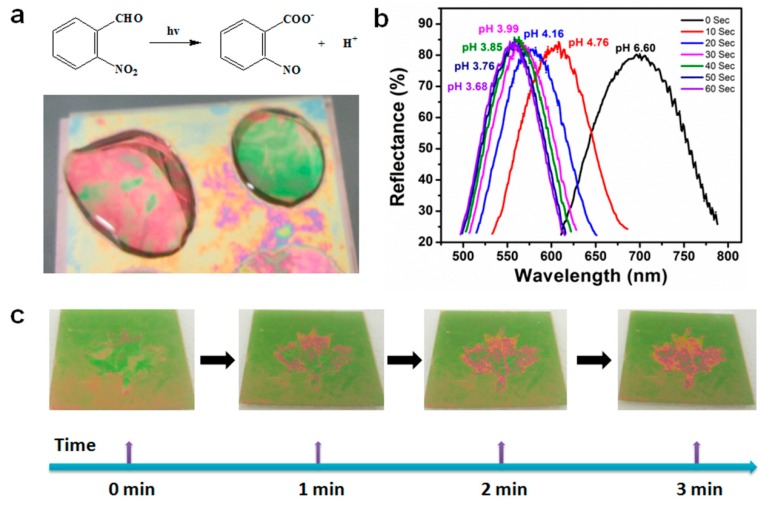
(**a**) Schematic depiction of the photoacid generation process upon exposure to UV light, and a pH responsive etalon that changes color upon light exposure and acid generation; (**b**) The dependence of the device reflectance spectrum at different pH values that were changed by UV exposure; (**c**) Photographs of patterned etalons (maple leaf pattern is pH responsive) after the indicated irradiation times. Reproduced with permission from the authors of [70], Copyright ^©^ 2014, Royal Society of Chemistry.

Etalon-based systems were also prepared that are capable of changing their optical properties in response to light by employing a photoacid combined with pH responsive microgels [70]. Specifically, a photoacid is a molecule that is capable of generating protons when exposed to UV irradiation, which can decrease the pH of a solution. Figure 17 shows the relationship between UV irradiation times, pH change and wavelength shift that also yields a visible color change—these results were achieved using the photoacid o-nitrobenzaldehyde (o-NBA). The color of this device could be visibly changed in less than 3 min. Light responsivity can easily be initiated/stopped by simply switching the excitation source on/off, while the magnitude of the response can be tuned by modulating the excitation source intensity, and/or wavelength. 

**Figure 18:**
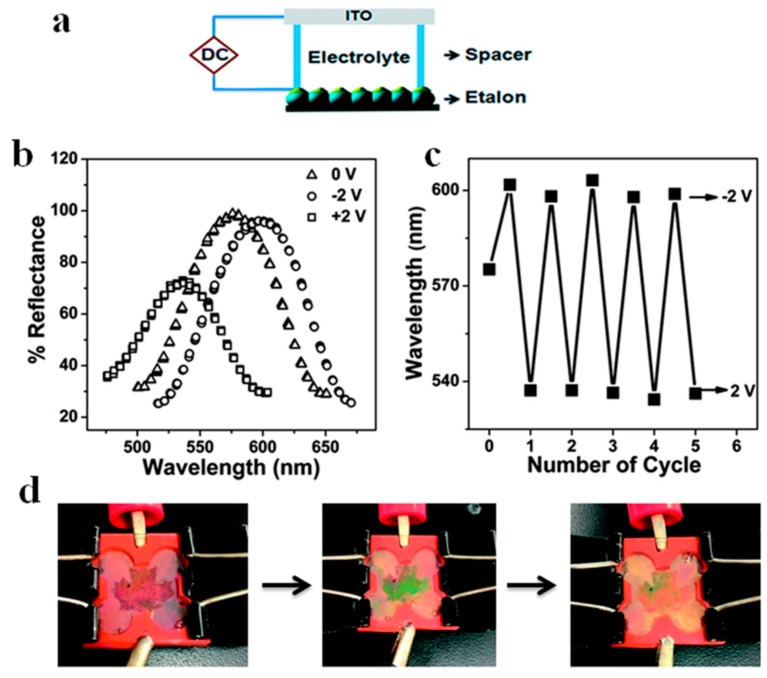
(**a**) Schematic of the experimental setup; (**b**) Reflectance spectra collected from an etalon after the application of the indicated voltages; (**c**) Final peak positions after application of the indicated potentials to the etalon over many cycles; (**d**) Photographs of a patterned etalon in an electrochemical cell at: 0 V, 2 V, −2 V, from left to right. Reproduced with permission from the authors of [59], Copyright ^©^ 2014, Royal Society of Chemistry.

Etalons that were sensitive to the application of an electric field were also fabricated and tested, and the response is shown in Figure 18. In this study, by applying a certain voltage (~3 V), a pH responsive etalon could exhibit visible and reversible color changes [59]. This is due to the hydrolysis of water at the applied potential, which subsequently changes the pH of the environment. Hence, if pH responsive etalons were exposed to this system, the etalon optical properties will likewise depend on applied electrical potential. The peak shift is related to the potential applied as shown in Figure 18b, and this phenomenon is completely reversible. We show that the etalon’s optical properties (color) are stable for many hours, until an appropriate potential is applied to bring the solution pH back to its initial value. In this example, we constructed an etalon with a maple leaf pattern that was pH responsive—the color of the maple leaf changed when an electrical potential was applied, and was reversible over many cycles, as shown in Figure 18d.

**Figure 19:**
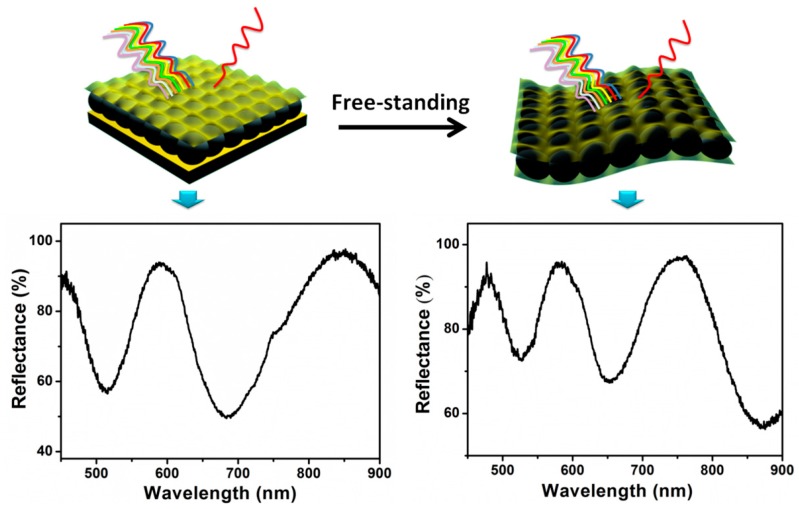
(**top**) Poly (*N*-isopropylacrylamide-co-acrylic acid) (pNIPAm-co-AAc) microgel-based free-standing etalons that exhibit (**bottom**) optical properties similar to substrate bound devices. Reprinted with permission from the authors of [71], Copyright ^©^ 2014, Royal Society of Chemistry.

While we have shown that microgel-based etalons can be fabricated on various planar substrates, there are some substrates that are difficult to coat with etalons, e.g., curved surfaces, rods, and tubes. Furthermore, for our sensing and monitoring efforts, it is advantageous to fabricate devices that can be adhered to skin. To achieve this, etalons could be fabricated on planar substrates containing a previously adsorbed sacrificial layer that could be easily dissolved and the etalon desorbed from the surface. The desorbed etalon can then be adhered to any other substrate as needed. To demonstrate that this is possible, we fabricated free-standing pNIPAm-co-AAc microgel-based etalons that exhibit high quality optical properties, which are capable of being transferred to multiple substrates, as shown in Figure 19. The etalon was fabricated on a solid support that was coated with a sacrificial polymer layer, which was generated by the layer-by-layer self-assembly technique. Therefore, the etalon could be easily removed from the solid substrate upon dissolution of the sacrificial layer. The desorbed etalons exhibit similar optical properties to the substrate adhered etalons, and retain their pH and temperature responsivity. This free-standing optical device will open new applications for sensing in environments that cannot tolerate the planar etalon geometry. Furthermore, the devices can be adhered to skin for real-time monitoring of human/animal health [71]. 

**Figure 20:**
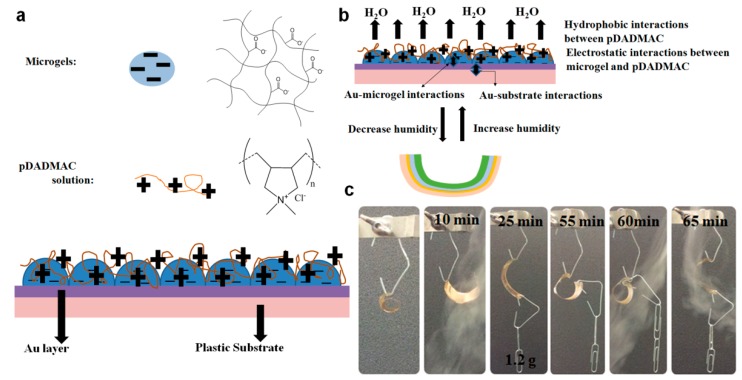
(**a**) Humidity sensor devices were constructed by depositing a single layer of pNIPAm-co-AAc microgels on a flexible plastic substrate (bottom layer) coated with an Au/Cr layer. After adding a solution of pDADMAC on top of the layer, a strong electrostatic interaction between the negatively charged microgels and the positively charged pDADMAC was formed; (**b**) The humidity sensing mechanism of this device. The pDADMAC layer resolvates or desolvated with the humidity change, and cause the device unbends or bend. This bending/unbending mechanism is completely reversible over many cycles; (**c**) A small curled substrate was hung from an arm and cycled between low and high humidity. Reprinted with permission from the authors of [72], Copyright ^©^ 2014, WILEY-VCH Verlag GmbH & Co. KGaA, Weinheim.

Furthermore, we recently developed polymer-based artificial muscles, which can act as sensing devices by utilizing responsive microgel and polymer layers [72]. The structure of this device is shown in Figure 20. As can be seen, negatively charged microgels were deposited on a Au-coated plastic substrate followed by the deposition of a solution of the cationic polyelectrolyte, poly(diallyldimethyl ammonium chloride) (pDADMAC). The assembly was then allowed to dry, and the device subsequently bends. The bending was due to the strong adhesion of the pDADMAC layer to the flexible substrate (through electrostatic interaction with the microgels), and it’s contraction upon drying. When the pDADMAC layer contracts upon drying the flexible substrate must bend. If the environmental humidity is increased, the pDADMAC layer rehydrates, and the device unbends, as shown schematically in Figure 20b. This bending/unbending mechanism is completely reversible over many cycles. We further showed that the device could be used to lift weights, as can be seen in Figure 20c and can move components around by lifting and dropping components in a humidity dependent fashion [72]. 

**Figure 21:**
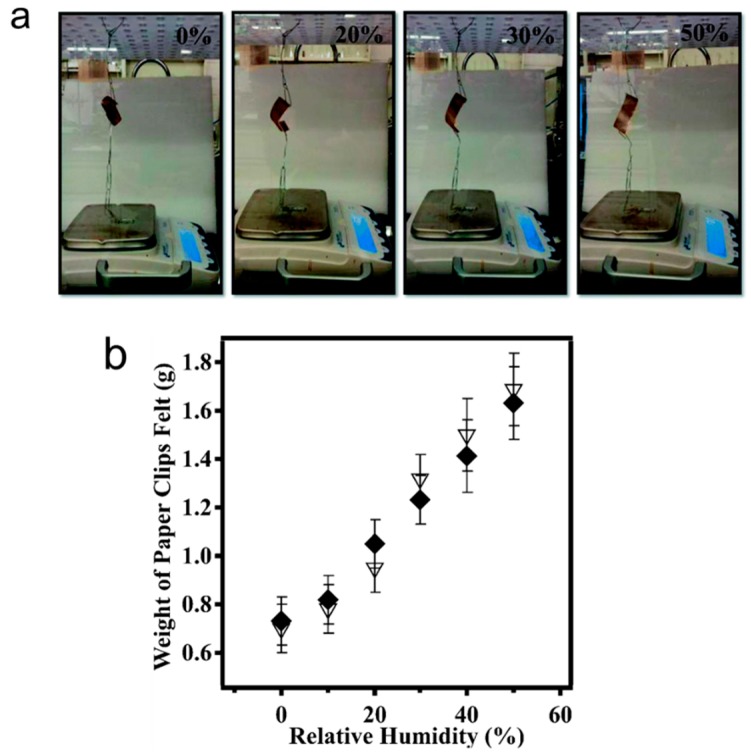
(**a**) Polymer-based actuator hanging in the humidity controlled chamber with paperclips attached, which were rested on the pan of a top loading balance. (**b**) The measured mass of the paperclips on the balance pan as a function of (♦) increasing and (∆) decreasing humidity. Reprinted with permission from the authors of [73], Copyright ^©^ 2014, Royal Society of Chemistry.

Furthermore, we demonstrated that the ability of the devices to lift masses can be used for sensing applications [73]. Firstly, the device and paperclips (used as weights) were added to a humidity-controlled chamber along with a top loading balance. The paperclips were allowed to rest on the balance, which recorded a mass. A photograph of the setup can be seen in Figure 21a. Initially, the chamber was held at 0% relative humidity, which caused the device to be completely curled up—this resulted in a relatively low mass on the balance. When the relative humidity was increased from 0% to 10% (usually takes ~30 min to fully stabilize) the device opened up, which allowed more of the paperclip mass to be added to the balance pan resulting in a concomitant increase in the measured mass. As the chamber humidity was incrementally increased, the device opened up, subsequently adding more of the paperclip mass to the balance pan. At 50% relative humidity, the device was completely open, bringing the mass to a maximum. The complete response of the device over the whole humidity range is shown in Figure 21b. 

## 4. Conclusions

We have briefly reviewed a few key examples of the use of responsive polymer-based photonic materials as sensors. The sensing mechanisms primarily depended on the responsive polymers changing the spacing between the PM/PC array elements, yielding the observed change in optical properties. While this is the case, changes in the lattice effective refractive index can also yield changes in their optical properties. PNIPAm microgel-based devices were subsequently introduced, and their use as sensors detailed, with a focus on pH, glucose, protein, and DNA sensing. We conclude that pNIPAm microgel-based devices have a lot of promise for sensing applications, although more work is needed to bring the technology to the market. The first challenge is the limited tunability of the PMs optical properties, which limits sensitivity; although, many examples in this review do show tunability over large wavelength ranges. Another limitation is response time; many examples in this review depend on diffusion to yield a response, which is a slow process. Yet another limitation is building reusability into the sensors; this could be addressed by exploiting the weakening of interactions with *T*, pH, and ionic strength to break signal-causing interactions to regenerate sensors. Despite the limitations, the foundations for building useful PM/PC-based sensors has been laid and further tuning of the devices can alleviate the above-mentioned drawbacks. Furthermore, new research is leading to a new understanding of how PM/PC-based sensors can be generated, which will lead to a new generation of PM/PC-based sensor technology.
